# Suicide-Related Knowledge and Attitudes among a Sample of Mental Health Professionals

**DOI:** 10.3390/ijerph18168296

**Published:** 2021-08-05

**Authors:** Denise Erbuto, Isabella Berardelli, Salvatore Sarubbi, Elena Rogante, Alice Sparagna, Gaia Nigrelli, David Lester, Marco Innamorati, Maurizio Pompili

**Affiliations:** 1 Department of Neurosciences, Mental Health and Sensory Organs, Faculty of Medicine and Psychology, Suicide Prevention Centre, Sant’Andrea Hospital, Sapienza University of Rome, 00100 Rome, Italy; denise.erbuto@gmail.com (D.E.); isabella.berardelli@uniroma1.it (I.B.); alice.sparagna@hotmail.it (A.S.); gaianigrelli@yahoo.it (G.N.); 2 Department of Human Neuroscience, Sapienza University of Rome, 00100 Rome, Italy; salvatore.sarubbi@uniroma1.it; 3 Department of Psychology, Sapienza University of Rome, 00100 Rome, Italy; elena.rogante@uniroma1.it; 4 Psychology Program, Stockton University, Galloway, NJ 08205, USA; david.lester@stockton.edu; 5 Department of Human Sciences, European University of Rome, 00100 Rome, Italy; marco.innamorati@unier.it

**Keywords:** suicide, knowledge, attitudes, prevention

## Abstract

Inadequate knowledge of the potential signs and risk factors of suicide negatively affects the ability of healthcare professionals to recognize patients at risk of suicide. The principal aim of the present study is to assess the attitudes and knowledge about suicide in a large sample of mental health professionals. We examined the relationship between Suicide Knowledge and Skills Questionnaire items and the experience of a patient dying by suicide. We also examined whether various healthcare professionals respond differently to the items of the Impact of a Patient’s Suicide on Professional and Personal Lives Scale. Results demonstrated that healthcare professionals who had experienced a patient suicide reported greater skills than professionals who had not experienced a patient suicide. However, 44% of professionals who had experienced a patient suicide felt that they did not have adequate training on this particular issue. Among those who had experienced a patient suicide, there was an increased tendency to hospitalize patients with suicide risk and an increased use of collegial consultation. Concerning personal emotions, healthcare professionals reported troubled relationships with family members and friends and the loss of self-esteem. In conclusion, better knowledge and attitudes about suicide are necessary for suicide-prevention strategies.

## 1. Introduction

The knowledge of how to identify and manage suicidal patients is important in suicide prevention. Training a range of professionals, not exclusively healthcare professionals, is an essential strategy in suicide prevention [1,2], and evidence suggests that training is necessary to increase knowledge about suicide in healthcare professionals [3,4,5,6]. Several studies have highlighted the fact that only half of the mental health trainees had received very limited training on suicide during their education [7]. Workshops proved to be effective for improving knowledge and shifting attitudes [8], although appropriate training, without supervised practice, does not necessarily imply that professionals obtain essential skills to deal with suicidal patients [9]. Moreover, patients at risk of suicide often visit primary care services rather than mental health professionals, thereby conferring an important role for primary care physicians for suicide assessment and prevention [10]. It has been reported that contact with primary healthcare services was highest in the year before suicide, with an average contact rate of 80%, while at one month the average rate was 44% [11]. Inadequate knowledge of the potential signs and risk factors of suicide negatively affects the ability of healthcare professionals to recognize patients at risk of suicide [12,13]. Improved knowledge and attitudes about suicide may allow healthcare professionals to better identify patients at risk, increasing suicide-prevention efforts. We define attitudes as a set of emotions, beliefs, and behaviors toward a particular object, person, thing, or event [14,15].

A patient’s suicide is not a rare event for healthcare professionals. At least half of all psychiatrists may have a patient who dies by suicide, and most professionals with previous experience can expect another suicide [16]. A patient’s suicide usually has a significant impact on healthcare professionals, affecting them on both a professional and a personal level [17]. The suicide of a patient can increase awareness of the various factors involved in suicide risk, creating anxiety, guilt, and high levels of stress [16]. Moreover, healthcare professionals often display negative attitudes toward patients with suicidal behaviors [18]. On the other hand, a patient’s suicide can offer an opportunity for professional growth, providing insight on personal competencies and encouraging consultations [19]. Regardless, healthcare professionals could benefit from improved knowledge about suicide, and training in suicide prevention may be necessary.

Based on the hypothesis that the attitudes of healthcare professionals toward people who died by suicide and their knowledge about suicide risk influence suicide-prevention strategies [20,21], the principal aim of the present study is to assess knowledge and attitudes about suicide in a large sample of mental health professionals. For this purpose, we examined the relation between Suicide Knowledge and Skills Questionnaire items [22] and the experience of a patient’s suicide. In addition, we also examined whether various healthcare professionals (i.e., physicians, psychologists, nurses) respond differently to the items of the Impact of a Patient’s Suicide on Professional and Personal Lives Scale.

## 2. Materials and Methods

### 2.1. Participants

A total of 670 healthcare professionals (physicians, psychologists, nurses, and educators) and medical and psychology students participated in the study (Table 1). Out of the 670 participants, 511 were female and the average was 44.89 years (SD = 13.81). Subjects were recruited during the 16th International Symposium on Suicidology and Public Health, which occurred in Rome in 2018. Approximately 26.7% of participants were physicians, 32.8% were psychologists, 14.2% were nurses, 15.7% were students, 4.3% were educators, and 5% were other mental health professionals (Table 1). Participants were invited to complete the questionnaire based on their skills and knowledge about suicide and the impact of a patient suicide on their lives. Approximately 250 healthcare professionals did not participate in the study Participation was voluntary and anonymous, and researchers and those associated with the project did not know the participants’ identities. Therefore, the information collected did not contain any identifiable information, and there was no risk of attributing data to particular individuals. All participants received a comprehensive explanation of the study procedures and goals, consistent with Helsinki’s Declaration. The project was submitted to the university department (Department of Neurosciences, Mental Health and Sensory Organs), which provided approval. 

### 2.2. Measures

We administered a socio-anamnestic questionnaire to all participants that included data on age, gender, and profession. The questionnaire also included a section reserved for professionals who had experienced a patient suicide.

The Suicide Knowledge and Skills Questionnaire (SKSQ) [22] is a 13-item questionnaire to assess knowledge about suicidal behaviors and perceived competence in managing suicidal patients. The questionnaire includes two scales: the suicide knowledge subscale with nine true or false sentences and the suicide skills subscale that estimates how confident healthcare professionals are in their training, skills, and supervision when managing suicidal patients. Responses are on a 5-point Likert scale that ranges from completely agree to disagree completely. Previous studies [22,23] only reported statistics on internal consistency and did not evaluate further the psychometric properties of the SKSQ. Both Smith et al. [22] and Silva et al. [23] reported low internal consistency for the suicide knowledge subscale (Cronbach’s alpha between 0.40 and 0.50), and acceptable internal consistency for the suicide skills confidence subscale (Cronbach’s alpha between 0.81 and 0.84); the low internal consistency of the suicide knowledge subscale was associated with the miscellaneous nature of the items [22,23]. Supporting the validity of the SKSQ as a measure of suicide-related knowledge and perceived skills, results from Smith et al. [22] suggest that scores on both subscales are able to discriminate between health professionals with specific training in suicide prevention and those who do not have any training. In the present sample, Cronbach’s alphas were 0.44 for the suicide knowledge subscale and 0.84 for the suicide skills confidence subscale. However, we did not treat the SKSQ as a psychometric scale (e.g., we did not calculate and interpret total scores as an index of a latent construct), and we analyzed only single items.

A self-report scale to assess the impact of a patient’s suicide on healthcare professionals’ professional and personal lives [24] was administered only to professionals who had experienced a patient suicide. This scale included 18 items that focused on personal life (e.g., personal feelings and difficulties with family members and friends) and professional life (e.g., changes in clinical practice and relationships with colleagues and supervisors). Responses were on a 7-point Likert scale with 1 = minimum impact and 7 = maximum impact. The authors [24,25] did not develop the items as part of a psychometric scale but used them only as part of a survey and did not perform any analysis to study the validity or reliability of the questionnaire. In the present sample, Cronbach’s alpha was 0.89. In line with the original studies [24,25], we did not use total scores and analyzed only responses to single items.

To analyze the results, we converted the 5-point Likert scale responses to the suicide skills subscale and the 7-point Likert scale responses to the Impact of a Patient’s Suicide on Professional and Personal Lives Scale to a 3-point response range. In the first questionnaire, we merged the responses “strongly disagree” and “disagree” and the responses “agree” and “strongly agree”, while in the second questionnaire we considered responses from 1 to 3 as “disagree”, responses from 5 to 7 as “agree”, and response 4 as “neither”. Since the distribution of the scores was non-normal, non-parametric statistics were used.

## 3. Results

Descriptive statistics are shown in Table 1. On average, participants had performed their job for 19.78 (±11.50) years. Over a third of the participants (37.31%) had experienced the suicide of a patient during their career. 

Examining the relationship between items of the suicide skills subscale and experience of patient suicide, those who had never experienced a patient suicide were less confident about their training and skills for handling suicidal patients (see Table 2). They were less likely to agree with the statements: “I have received the training I need to engage and assist those with suicidal desire”, “I have the skills I need to engage those with suicidal desire and/or intent”, and “I am comfortable asking direct and open questions about suicide”. However, a large percentage of those who had experienced a patient’s suicide felt that they did not have enough training or skills: 44.3% did not believe they had received the necessary training to engage and assist those with suicidal desire, 25.1% were not confident about their skills to engage patients with suicidal ideation, and 18.9% were not comfortable asking about suicide (Table 2). To study whether participants’ years of professional experience could affect the relationship between the experience of patients’ suicide and the participants’ suicide skills, we performed a loglinear analysis with the experience of a patients’ suicide as a dependent variable and variables significant at the bivariate analyses (i.e., training, skills, and comfort) and years of experience as independent variables. The model fitted the data adequately (Likelihood Ratio χ^2^ = 0.93, *p* = 0.63). The experience of a patient’s suicide was significantly associated with comfort in asking about suicide (Odds Ratio = 2.19, *p* = 0.001). In contrast, having enough training (Odds Ratio = 1.26, *p* = 0.49) or skills (Odds Ratio = 1.38, *p* = 0.31), and years of experience (Odds Ratio = 1.02, *p* = 0.73) were not significantly associated with the experience of a patients’ suicide. What is noteworthy here is, regardless of the experience of a patient suicide, a large proportion of the participants did not feel that they had the training or skills to help suicidal patients, and 60% of the total sample did not feel that they had the support and supervision that they needed to engage with and assist those with suicidal desires.

There were no significant associations between knowledge about suicide and the experience of a patient suicide (see Table 3).

Regarding the impact of a patient’s suicide, there were significant differences by profession for 7 of the 18 items (see Table 4). Regarding the impact of a patient’s suicide, 28.8% of physicians, 37.7% of psychologists, and 41.4% of nurses reported an increased tendency to hospitalize patients. Approximately 35.8% of physicians, 49.2% of psychologists, and 71.9% of nurses used collegial consultations or peer consultations. Approximately 7.1% of physicians, 6.9% of psychologists, and 25.0% of nurses experienced problems in relationships with friends, while 4.0% of physicians, 14.0% of psychologists, and 28.1% of nurses experienced problems with family. Loss of self-esteem was reported by 13.7% of physicians, 13.3% of psychologists, and 28.1% of nurses. Finally, 8.2% of physicians, 8.6% of psychologists, and 25.0% of nurses experienced emotional numbness (Table 4).

## 4. Discussion

The principal aim of the present study was to assess self-reported attitudes and knowledge about suicide in a large sample of mental health professionals. For this purpose, we examined the relationship between the Suicide Knowledge and Skills Questionnaire items and the experience of a patient’s suicide. In addition, we also examined the association between items of the Impact of a Patient’s Suicide on Professional and Personal Lives Scale and different healthcare professions.

We found a significant relationship between the experience of a patient’s suicide and items on the SKSQ. A large proportion of the participants did not feel that they had the training or skills to help suicidal patients, and 60% of the total sample did not feel that they had the support and supervision needed to engage with and assist those with suicidal desires. However, the results indicated that healthcare professionals who had experienced a patient suicide reported greater skills than professionals who had not experienced a patient suicide. Feeling comfortable asking about suicide was still significant in multivariate analysis while controlling for years of professional experience. These findings can be explained by the fact that healthcare professionals usually receive more training in suicide prevention after a patient suicide. Several psychiatric services offer psychological support after a patient’s suicide, and healthcare professionals who had experienced a patient’s suicide learned from that experience how to better manage suicide risk [26,27]. Healthcare professionals who had experienced a patient’s suicide reported administering improved suicide risk assessments, felt better able to ask patients questions about suicidal ideation, and were more aware of suicide risk factors [28]. 

Previous studies have shown that healthcare professionals who worked with suicidal patients had reported a better knowledge of suicide and were more likely to receive suicide-related training [29,30]. Thus, experiencing a patient suicide may prompt healthcare professionals to seek further training, and this training may increase the willingness of healthcare professionals to treat patients at risk of suicide [23]. 

However, we did not find a significant difference for the item “I have the support/supervision I need to engage and assist those with suicidal desire” between professionals who had experienced a patient suicide and those who had not. Both groups felt that they did not have the necessary support to manage suicidal patients. In particular, 44% of professionals who experienced a patient suicide felt that they did not have the proper training on this particular issue. Our results support previous papers that have demonstrated that only a small percentage of healthcare professionals consider themselves sufficiently trained to work with patients at risk of suicide [31,32]. In a study by Samuelsson et al. [33] on 191 psychiatric nurses, only 25% considered themselves sufficiently trained for this work. In another study with a sample of 300 psychiatric staff, only 44.3% considered themselves sufficiently trained [31]. 

Finally, we examined the association between the Impact of a Patient’s Suicide on Professional and Personal Lives Scale and the different healthcare professions. The results showed a significant relationship between the profession and “increased tendency to hospitalize,” “increased use of collegial consultation”, “increased use of peer consultation”, “disturbed relationships with friends”, “loss of self-esteem”, “disturbed relationships with family”, “emotional numbness”, with nurses reporting the more negative impact than did psychiatrists and psychologists. Compared to other professional figures, the more negative impact reported by nurses might be explained by their broad education background, not necessarily involving mental health issues and perspectives. Overall, our results show that improved knowledge and improved attitudes toward suicide are important factors for suicide-prevention strategies.

A study conducted by Takahashi et al. [34], in fact, has analyzed the exposure to patients’ suicide and the consequences on nurses in terms of stress. The results have shown that the absence of formal systems to deal with the psychological aftermaths of such an event causes significant distress and a higher burden on nurses. 

Moreover, after a patient suicide, there was an increased tendency to hospitalize patients with suicide risk and an increased use of collegial consultation. Concerning personal emotions, healthcare professionals reported troubled relationships with family and friends and a loss of self-esteem, confirming the personal and professional consequences of a patient’s suicide on healthcare professionals [35]. 

This study had several limitations. First, the healthcare professionals involved in the study belonged to different professional groups. Some groups interviewed had a small sample size. Second, the participants attended a symposium on suicide and may not represent all healthcare professionals. Finally, the perception of being sufficiently trained is not an objective measure since individuals may misjudge their competence.

## 5. Conclusions

In conclusion, this study investigated an issue of public health interest: the preparation of healthcare professionals for assessing and treating suicidal patients. Our results highlight the emotional and professional impact that a patient suicide can have on healthcare professionals. Our results demonstrated that a large proportion of the participants did not feel that they had the training or skills to help suicidal patients. Overall, our results show that increased knowledge and improved attitudes about suicide are important factors for suicide-prevention strategies. As suggested in previous studies, developing suicide-prevention campaigns and training seems essential for increasing the knowledge of healthcare professionals about suicide and people at risk of suicide, their family members, and people bereaved by suicide [36,37]. Furthermore, offering programs and training on suicide are important for other professionals, including school personnel, researchers, and policymakers, to improve prevention strategies to reduce suicide risk [1].

## Figures and Tables

**Table 1:** Characteristics of the sample.

Participants	Percentage (%)
Sex	
Male	23.6
Female	76.3
Age-M ± SD (years)	44.89 ± 13.81
Profession	
Physician	26.7
Child neuropsychiatry	3.0
Psychiatry	17.8
Neurology	0.6
Psychotherapy	7.3
Other specialties	1.5
Psychologist	32.8
Nurse	14.2
Educator	4.3
Resident	1.3
Student	15.7
Other	3.7
Job affiliation	
Outpatient clinic	40.0
Inpatient clinic	12.3
Other	26.0
Unknown	21.7
Profession-number of years—M ± SD	19.78 ± 11.50
Past experiences of patients’ suicide	37.31
Number of suicides experienced by the professional (mean)	2.21

**Table 2:** Contingency table suicide skills subscale items: past experiences of patients’ suicide.

Suicide Skills Subscale Items		No Patient Suicide Reported		Past Patient Suicide			
		*N*	%	*N*	%	χ^2^	*p*
I have received the TRAINING I need to engage and assist those with suicidal desire	Disagree	210	62.9	104	44.3	23.02	<0.001
	Undecided	55	16.5	43	18.3		
	Agree	69	20.7	88	37.4		
I have the SKILLS I need to engage those with suicidal desire and/or intent	Disagree	179	53.1	59	25.1	53.26	<0.001
	Undecided	85	25.2	67	28.5		
	Agree	73	21.7	109	46.4		
I am comfortable asking direct and open questions about suicide	Disagree	153	45.4	45	18.9	55.28	<0.001
	Undecided	58	17.2	32	13.4		
	Agree	126	37.4	161	67.6		
I have the SUPPORT/SUPERVISION I need to engage and assist those with suicidal desire	Disagree	209	62.0	133	56.7	1.86	0.39
	Undecided	37	11.0	32	13.6		
	Agree	91	27.0	70	29.8		

**Table 3:** Suicide knowledge by experience with a suicide.

Items of Suicide Knowledge Questionnaire		No Patient Suicide Reported		Past Patient Suicide			
		*N*	%	*N*	%	χ^2^	*p*
**1.** Few people want to kill themselves (F)	UNCORRECT RESPONSE	116	34.0	102	40.8	2.85	0.09
	CORRECT RESPONSE	225	66.0	148	59.2		
**2.** Youth ages 10–24 have a significantly greater risk of suicide than individuals aged 65 or older (F)	UNCORRECT RESPONSE	209	61.3	142	56.8	1.21	0.27
	CORRECT RESPONSE	132	38.7	108	43.2		
**3.** The rate of suicide among those with severe mental illness is 6 times the general population (T)	UNCORRECT RESPONSE	172	50.4	126	50.4	0.00	0.99
	CORRECT RESPONSE	169	49.6	124	49.6		
**4.** If a person is serious about suicide. there is little that can be done to prevent it (F)	UNCORRECT RESPONSE	89	26.1	77	30.8	1.58	0.21
	CORRECT RESPONSE	252	73.9	173	69.2		
**5.** If you talk to a client about suicide. you may inadvertently give them permission to seriously consider it (F)	UNCORRECT RESPONSE	80	23.5	59	23.6	0.00	0.97
	CORRECT RESPONSE	261	76.5	191	76.4		
**6.** Depression indicates a suicide risk (T)	UNCORRECT RESPONSE	36	10.6	37	14.8	2.40	0.12
	CORRECT RESPONSE	305	89.4	213	85.2		
**7.** Suicide is always unpredictable (F)	UNCORRECT RESPONSE	98	28.7	78	31.2	0.42	0.52
	CORRECT RESPONSE	243	71.3	172	68.8		
**8.** Suicidal people want to die (F)	UNCORRECT RESPONSE	104	30.5	81	32.4	0.24	0.62
	CORRECT RESPONSE	237	69.5	169	67.6		
**9.** Individuals with Borderline Personality Disorder frequently discuss or gesture suicide but do not really intend to kill themselves; instead, they intend to provoke or manipulate others (F)	UNCORRECT RESPONSE	204	72.7	176	70.4	0.36	0.53
	CORRECT RESPONSE	93	27.3	74	29.6		

**Table 4:** Impact of a patient suicide on the professional and personal lives of healthcare professionals.

ITEMS		Physician		Psychologist		Nurse		Other		χ^2^	*p*
		*N*	%	*N*	%	*N*	%	*N*	%		
**1.** Increased attention to legal aspect of practice	DISAGREE	34	31.8	20	31.3	5	16.1	3	18.8		
	NEITHER	9	8.4	4	6.3	4	12.9	2	12.5	4.69	0.58
	AGREE	64	59.8	40	62.5	22	71.0	11	68.8		
**2.** Increased tendency to hospitalize	DISAGREE	55	52.9	34	55.7	12	41.4	3	23.1		
	NEITHER	19	18.3	4	6.6	5	17.2	1	7.7	13.01	0.04
	AGREE	30	28.8	23	37.7	12	41.4	9	69.2		
**3.** More conservative patient selection	DISAGREE	75	74.3	49	81.7	23	74.2	11	73.3		
	NEITHER	10	9.9	7	11.7	3	9.7	0	0.0	6.38	0.38
	AGREE	16	15.8	4	6.7	5	16.1	4	26.7		
**4.** Increased focus on suicide cues	DISAGREE	17	16.2	8	12.3	2	6.1	2	12.5		
	NEITHER	8	7.6	7	10.8	0	0.0	0	0.0	8.11	0.23
	AGREE	80	76.2	50	76.9	31	93.9	14	87.5		
**5.** Increased concerns with death issues	DISAGREE	21	20.2	21	32.8	4	12.5	3	18.8		
	NEITHER	18	17.3	6	9.4	4	12.5	4	25.0	8.83	0.18
	AGREE	65	62.5	37	57.8	24	75	9	56.3		
**6.** Increased use of collegial consultation	DISAGREE	46	43.4	19	30.2	7	21.9	3	23.1		
	NEITHER	22	20.8	13	20.6	2	6.3	3	23.1	15.11	0.02
	AGREE	38	35.8	31	49.2	23	71.9	7	53.8		
**7.** More conservative record keeping	DISAGREE	44	42.3	24	38.7	8	25.8	4	26.7		
	NEITHER	16	15.4	9	14.5	3	9.7	0	0.0	9.32	0.16
	AGREE	44	42.3	29	46.8	20	64.5	11	73.3		
**8.** Increased use of peer consultation	DISAGREE	41	38.3	13	21.0	2	6.3	4	25.0		
	NEITHER	19	17.8	11	17.7	2	6.3	2	12.5	21.89	0.00
	AGREE	47	43.9	38	61.3	28	87.5	10	62.5		
**9.** Disturbed relationships with colleagues	DISAGREE	80	80.0	50	83.3	22	68.8	12	80.0		
	NEITHER	8	8.0	3	5.0	3	9.4	1	6.7	3.26	0.78
	AGREE	12	12.0	7	11.7	7	21.9	2	13.3		
**10.** Disturbed relationships with friends	DISAGREE	86	86.9	54	93.1	21	65.6	13	86.7		
	NEITHER	6	6.1	0	0.0	3	9.4	2	13.3	18.55	0.00
	AGREE	7	7.1	4	6.9	8	25.0	0	0.0		
**11.** Loss of self-esteem	DISAGREE	86	84.3	43	71.7	20	62.5	11	73.3		
	NEITHER	2	2.0	9	15.0	3	9.4	1	6.7	14.66	0.02
	AGREE	14	13.7	8	13.3	9	28.1	3	20.0		
**12.** Dreams related to suicide	DISAGREE	82	82.8	50	89.3	27	87.1	15	100.0		
	NEITHER	6	6.1	1	1.8	2	6.5	0	0.0	5.00	0.54
	AGREE	11	11.1	5	8.9	2	6.5	0	0.0		
**13.** Disturbed relationships with family	DISAGREE	89	89.9	48	84.2	21	65.6	13	86.7		
	NEITHER	6	6.1	1	1.8	2	6.3	0	0.0	17.15	0.01
	AGREE	4	4.0	8	14.0	9	28.1	2	13.3		
**14.** Intrusive thoughts of suicide	DISAGREE	80	81.6	49	83.1	21	65.6	13	86.7		
	NEITHER	8	8.2	6	10.2	4	12.5	0	0.0	7.40	0.29
	AGREE	10	10.2	4	6.8	7	21.9	2	13.3		
**15.** Guilt	DISAGREE	63	60.0	38	61.3	16	50.0	9	60.0		
	NEITHER	15	14.3	7	11.3	4	12.5	2	13.3	2.03	0.92
	AGREE	27	25.7	17	27.4	12	37.5	4	26.7		
**16.** Anger	DISAGREE	59	57.8	28	44.4	14	43.8	8	50.0		
	NEITHER	13	12.7	10	15.9	3	9.4	2	12.5	5.06	0.54
	AGREE	30	29.4	25	39.7	15	46.9	6	37.5		
**17.** Emotional numbness	DISAGREE	87	88.8	50	86.2	19	59.4	13	86.7		
	NEITHER	3	3.1	3	5.2	5	15.6	0	0.0	17.18	0.01
	AGREE	8	8.2	5	8.6	8	25.0	2	13.3		
**18.** Social withdrawal	DISAGREE	90	91.8	51	85.0	24	75.0	13	86.7		
	NEITHER	2	2.0	2	3.3	2	6.3	0	0.0	6.91	0.33
	AGREE	98	6.1	60	11.7	32	18.8	15	13.3		

## Data Availability

The datasets used and/or analyzed during the current study are available from the corresponding author on reasonable request.
